# Endogenous sex hormone levels are associated with the revised Framingham Stroke Risk Profile in postmenopausal women: a longitudinal study in a Swedish cohort

**DOI:** 10.1186/s12902-025-01841-3

**Published:** 2025-01-26

**Authors:** Kristin Ottarsdottir, Åsa Tivesten, Claes Ohlsson, Ying Li, Margareta Hellgren, Ulf Lindblad, Bledar Daka

**Affiliations:** 1 Family medicine, School of Public Health and Community Medicine, Institute of Medicine, Sahlgrenska Academy, University of Gothenburg, Box 454, Göteborg, 40530 Sweden; 2 Wallenberg Laboratory for Cardiovascular and Metabolic Research, Department of Molecular and Clinical Medicine, Institute of Medicine, Sahlgrenska Academy, University of Gothenburg, Gothenburg, Sweden; 3 Biostatistics, School of Public Health and Community Medicine, Institute of Medicine, Sahlgrenska Academy, University of Gothenburg, Gothenburg, Sweden; 4 Sahlgrenska Osteoporosis Centre, Centre for Bone and Arthritis Research, Department of Internal Medicine and Clinical Nutrition, Institute of Medicine, Sahlgrenska Academy, University of Gothenburg, Gothenburg, Sweden; 5 The Skaraborg Institute, Skövde, Sweden

**Keywords:** Sex hormones, Postmenopause, Stroke, Cardiovascular risk factors, Mass spectrometry

## Abstract

**Background:**

Endogenous sex hormones in postmenopausal women have been associated with risk of cardiovascular diseases. The aim of this study was to determine the association between endogenous sex hormones and the revised Framingham Stroke Risk Profile (rFSRP) in postmenopausal women.

**Methods:**

This is an observational cross-sectional study on the Vara-Skövde cohort, a Swedish population-based study for longitudinal surveillance of the development and progress of type 2 diabetes and hypertension. The participants were physically examined in 2002–2005 and sex hormones were analysed with liquid chromatography-tandem mass spectrometry assay (LC-MS/MS). Women who were **≥**55 years old, with estradiol levels below 20 pg/mL, not using hormonal therapy, and with no self-reported history of stroke, were included (*N* = 133). The outcome variable was rFSRP. Regression analyses of log-transformed rFSRP were fitted against levels of sex hormones (17-α-OH-progesterone, estrone, estradiol, progesterone, dihydrotestosterone, dehydroepiandrosterone, testosterone and androstenedione), adjusting for body mass index (BMI) or waist-to-hip ratio (WHR), C-reactive protein (CRP) and cholesterol level.

**Results:**

Levels of estrone and estradiol were positively associated with rFSRP in the crude model (estrone β = 0.208, 95% CI = 0.081;0.336, *P* = 0.002; estradiol β = 0.170, CI = 0.034;0.305, *P* = 0.015). Adjustments for BMI revealed significant positive associations between progesterone (β = 0.155 95% CI = 0.025;0.285, *P* = 0.020), estrone (β = 0.167, 95% CI = 0.037;0.297, *P* = 0.013) and 17-α-OH-progesterone (β = 0.146, 95% CI = 0.014; 0.277, *P* = 0.030) and rFSRP, and adjustments for WHR revealed a significant positive association between testosterone and rFSRP (β = 0.152, CI = 0.026;0.278, *p* = 0.018).

**Conclusions:**

Increase of estrone was associated with higher rFSRP, also in the fully adjusted model, whereas progesterone, 17-α-OH-progesterone and testosterone were significant only in the models adjusting for BMI and WHR respectively. Larger studies studying stroke events are warranted to confirm these findings.

**Clinical trial number:**

Not applicable.

**Supplementary Information:**

The online version contains supplementary material available at 10.1186/s12902-025-01841-3.

## Introduction

Stroke, ischaemic or haemorrhagic, is the second leading cause of death globally, causing about 6.55 million deaths in 2019 [1]. Although age is the strongest risk factor for developing stroke, high blood pressure, high body-mass index and high fasting glucose are other leading risk factors [1]. In postmenopausal women, abdominal adiposity, elevated triglycerides and cholesterol have been identified as particularly significant risk factors [2]. One way to assess the individual’s risk of developing stroke is to use a risk-scoring tool. The original Framingham Stroke Risk Profile, first introduced in 1991, presented an estimation tool for the probability of incident stroke in 10 years, taking age, systolic blood pressure, use of antihypertensive medications, presence of left-ventricular hypertrophy on electrocardiography, prevalent cardiovascular disease, smoking, atrial fibrillation and diabetes mellitus status into account [3]. Since stroke incidence has decreased, and the distribution of the risk factors has changed during the last decades, a revised version of the risk score profile (rFRSP) was launched in 2018 [4], that better predicts the current stroke risk compared to the old Framingham stroke risk profile. The revised risk score was developed based on the risk factor prevalence and hazard ratio for stroke incidence in the Framingham Heart Study during the past 20 years, and then validated in two different external study samples (REGARDS and the 3 cities study) [4].

Stroke incidence and mortality caused by stroke is lower in women than in men, which may, at least partially, be related to the actions of sex hormones [5]. In the Framingham study, over 5000 female participants were followed up for 56 years, revealing that women develop stroke on average 5 years later than men, and had a lower stroke incidence at all ages except for the age group 85 years old and older. However, due to their longer life expectancy, women had a higher lifetime risk of stroke [6]. During the menopausal transition, disadvantageous changes in body fat distribution and lipids occur, and stroke incidence and coronary heart disease incidence increase after menopause [7]. Previous studies have suggested that stroke risk increases with early menopause, but the literature is not consistent [8].

In the early postmenopausal period, endogenous sex hormones, especially estrogens from the ovaries, decreases by approximately 90%, and thereafter there is a further decline in estrogens [9]. Both progesterone and its hydroxylated form 17-α-OH-progesterone generally circulate in very low concentrations in postmenopausal women and the evidence about endogenous progesterone and 17-α-OH progesterone is scarce, especially in relation to cardiovascular health.

Liquid chromatography-tandem mass spectrometry (LC-MS/MS) is the gold standard for assessing sex hormones, due to its high precision even at very low hormone levels [10], however, many previous studies have used other assays than LC-MS/MS, and it is uncertain to what extent sex hormones play a role in the risk of developing stroke in postmenopausal women. The aim of this study has been to investigate the association between levels of endogenous sex hormones, and the revised Framingham Stroke Risk Profile in a cohort of postmenopausal women.

## Methods and subjects

### Study population

**Fig. 1:**
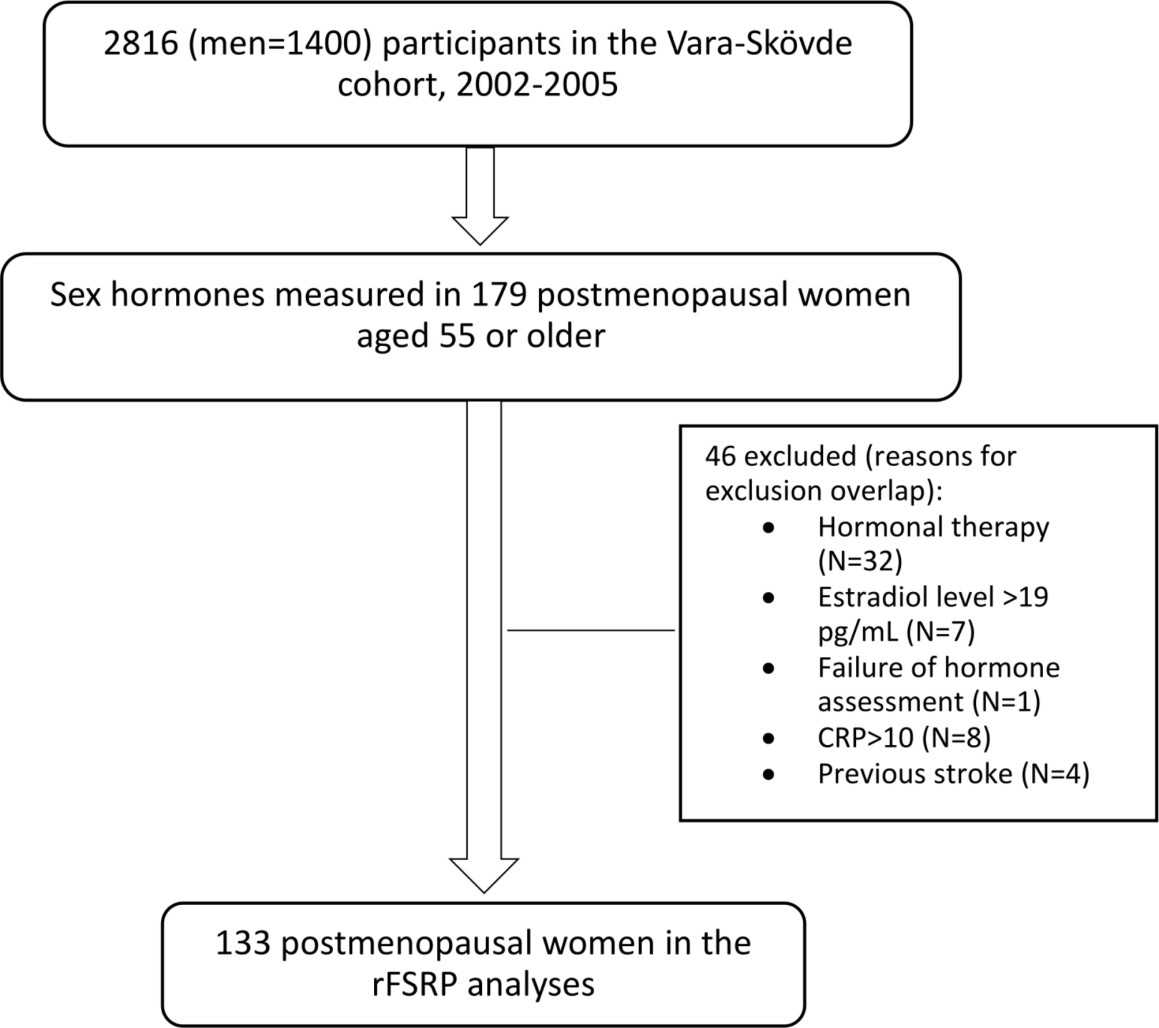
Study flowchart showing the inclusion process

The Vara-Skövde cohort [11] was first examined in 2002–2005. The study population was randomly selected from the population aged 30–74 in the census register and stratified by sex and by 5-year age groups. There was an intentional three-fold oversampling of participants aged 30–50 compared to participants 50 years old and older. The original aim of the cohort study was to investigate the development of hypertension and type 2 diabetes at an early stage and 2816 individuals participated (Men = 1400). A detailed description of the study design has been published elsewhere [11]. In the study population, sex hormones (17-α-OH-progesterone, estrone, estradiol, progesterone, dihydrotestosterone, dehydroepiandrosterone, testosterone and androstenedione) were analysed with a validated high-sensitivity liquid chromatography-tandem mass spectrometry assay [12] in 179 women, all of whom were 55 years old or older at visit 1. No self-reported information regarding menopause state was available, therefore only participants aged 55 years old or older and estradiol concentrations less than 20 pg/mL [13] were included, to limit the risk of including women before menopause. A total of 133 women were included in the present cross-sectional study (Fig. 1). All participants gave their written informed consent to participate, and the Regional Ethical Review Board in Gothenburg, Sweden approved the study (registration numbers Ö 199-01). The study adhered to the ethical principles of the Helsinki declaration.

### Physical examination

The study participants were examined by trained research nurses. Waist circumference was measured between the lowest rib margin and iliac crest. Body height was measured without shoes, and body weight was measured with a scale, without shoes and only light clothing on. Blood pressure was measured in supine and standing positions, after the participants had rested for 5 min. Two blood pressure measurements were made with one minute between, and the mean of these two results was recorded. Body mass index (BMI) was calculated by the body weight in kilograms divided by the square of the height in meters, and waist-to-hip ratio (WHR) was calculated. Information on medical history and medications was obtained by trained nurses and by using questionnaires [14, 15], and further information about smoking habits, medical history and medications was obtained from the participants. Previous history of stroke and cardiovascular disease was self-reported. The diagnoses of diabetes mellitus was defined based on World Health Organization (WHO) [16] (**≥**7.0 mmol/L of fasting plasma glucose at two separate occasions, or **≥**11.1 mmol/L of plasma glucose 2 h after an oral glucose load). Hypertension was defined as blood pressure **≥**140/90 mm Hg based on Joint National Committee (JNC7) [17] recommendations.

### Laboratory analyses

Fasting venous blood samples were drawn in the morning. All blood samples were immediately frozen at -82 °C. Serum concentrations of sex hormones (17-α-OH-progesterone, estrone, estradiol, progesterone, dihydrotestosterone, dehydroepiandrosterone, testosterone and androstenedione) were assessed from stored samples with a validated high-sensitivity liquid chromatography-tandem assay [12], which has a low intra- and interassay coefficients of variation, and has lower limits of quantification for estradiol, estrone and progesterone than many previously used LC-MS/MS assays [12]. The assay is described in detail in the study by Ohlsson et al. [12]. Lower limits of quantification were (in pg/mL) 20 for 17-α-OH-progesterone, estrone 0.5, estradiol 0.5, progesterone 5, dihydrotestosterone 13, dehydroepiandrosterone 250, testosterone 5 and androstenedione 5 pg/mL. One participant had value below LLOQ regarding estradiol, and two participants had values below LLOQ regarding progesterone, and one participant had value below LLOQ regarding DHT. These variables were set to the value of LLOQ for each hormone respectively.

High sensitivity CRP (hs-CRP, in mg/L)) serum concentrations were assessed with radio immune assay.

### Risk scores

The revised Framingham Stroke Risk Profile (rFSRP) was calculated for each participant based on the revised Framingham score formula presented by Dufouil et al. [4]. This risk score algorithm takes the following risk factors in account: age, current smoking, prevalent cardiovascular disease (excluding stroke), prevalent atrial fibrillation (AF), diabetes (DM), medication for hypertension (HRX) and systolic blood pressure. The equation used for females is:

*FSRP* (*t*) = 1 - *Sb* (*t*) ^exp(*L*−*M*)^, where *t* =10, S b[10] = 0.95911, M = 6.6170719, and L is the linear combination: L = 0.87938 * (Age/10) + 0.51127 *[if smoker]* − 0.03035 *[if CVD]* + 1.20720 *[if AF]* +0.39796 *[if Age* ≥ *65]* + 1.07111 *[if Age* < *65 and DM]* + 0.06565 *[if Age* ≥ 65 *and DM]* + 0.13085 *[if HRX]* + (0.11303 * (SBP–120)/10) *[if no HRX]* +(0.17234 * (SBP–120)/10) *[if HRX].*

Previous or prevalent cardiovascular disease (CVD) that is used in the algorithm for rFSRP was defined as self-reported angina pectoris, heart failure, or previous myocardial infarction.

### Statistical analyses

Multiple linear regression analyses were used with sex hormones as explanatory variables and rFSRP as the dependent variable. Due to the skewed distribution of the rFRSP, this variable was transformed into a natural logarithm to improve the model fit. Model 1 is the crude model without any adjustment. Model 2 included adjustments for body mass index (BMI) for the estrogens and waist-to-hip-ratio (WHR) for the androgens, since those variables are more associated with those respective hormones, as described in a previous study of this cohort [18]. Since inflammation and cholesterol levels may be correlated to both hormonal level and the outcome [19], model 3 included additional adjustments for high-sensitivity C-reactive protein (hsCRP) and total cholesterol level. It’s worth noting that the algorithm for calculating rFSRP inherently incorporates age as a parameter. In our previous work [18], we demonstrated that age only showed a significant correlation with testosterone levels. Thus, based on our data, age was not considered as a confounding variable and not included adjustment in the analysis. The number of participants in the analyses for rFSRP-score was 133.

As a sensitivity analysis, we conducted regression analyses after stratifying for the median age of 64 years old. Given that many of the sex hormones are both precursors and metabolites bivariate correlation analysis was performed to investigate the correlations between the 8 sex hormones in the cohort.

To explore the associations also when adjusting for sex hormone binding globulin an additional analysis was added including SHBG to the last model, and a separate analysis was also performed with SHBG as exposure variable.

The regression results are presented on a standardized SD scale, i.e. coefficients and CI are multiplied by SD. This standardization facilitates comparison of the results across different sex steroids. All statistical analyses were performed with R 4.3.1.

## Results

Baseline characteristics of the study population are shown in Table 1.

**Table 1:** Characteristics of the study population, *N* = 133

	Mean	SD
Age (years)	64.5	5.2
Revised Framingham Stroke Risk Profile (%)	3.5	2.7
	Geometric mean	95% CI
Systolic blood pressure (mmHg)	133.0	130.3; 135.8
Diastolic blood pressure (mmHg)	71.6	69.9; 73.2
BMI (kg/m2)	27.4	26.6; 28.2
WHR	0.8	0.8; 0.9
Sex hormone binding globulin (nmol/L)	69.9	64.5; 75.8
Total cholesterol (mmol/L)	5.1	4.9; 5.3
CRP (mg/L)	1.8	1.6; 2.1
Progesterone (pg/mL)	43.0	39.3; 47.0
17-a-OH-progesterone (pg/mL)	304.2	274.8; 336.8
Estrone (pg/mL)	22.3	20.8; 23.9
Estradiol (pg/mL)	4.2	3.8; 4.6
Testosterone (pg/mL)	231.5	213.3; 251.2
DHEA (pg/mL)	2382.1	2151.6; 2637.3
Dihydrotestosterone (pg/mL)	39.8	35.8; 44.4
Androstenedione (pg/mL)	560.5	519.8; 604.5
	N	%
Type 2 diabetes n (%)	15	11.3
Prevalent atrial fibrillation n (%)	0	0
Previous CVD n (%)	8	6.0
Hypertension n (%)	38	28.6

Abbreviations: BMI = body mass index, WHR = waist-to-hip ratio, CRP = C-reactive protein, DHEA = dehydroepiandrosterone

Mean age was 64.5 years old (SD 5.2 years, range 55–74 years old), mean BMI was 27.8 kg/m^2^ (SD 4.9 kg/m^2^) and 11.3% of the women had diabetes mellitus. The mean estimated 10-year risk for stroke was 3.5% (SD 2.7%) according to rFRSP. Due to the skewed distribution biomarkers and sex hormones were presented with the geometric mean and 95% confidence interval.

Significant associations between sex hormones and rFSRP were observed (Table 2*).*

**Table 2:** Endogenous sex hormones and the revised Framingham Stroke Risk Profile, results from linear regression model. *N* = 133

	β	2.5%CI	97.5%CI	*p*
Progesterone				
Model 1 Crude	0.094	-0.037	0.225	0.159
Model 2 (adjusted for BMI)	0.155	0.025	0.285	**0.020**
Model 3 (adjusted for BMI, CRP and cholesterol)	0.150	0.017	0.283	**0.027**
Model 3 + adjustment for SHBG	0.150	0.013	0.288	**0.032**
**17-α-OH-progesterone**				
Model 1 Crude	0.112	-0.024	0.248	0.106
Model 2 (adjusted for BMI)	0.146	0.014	0.277	**0.031**
Model 3 (adjusted for BMI, CRP and cholesterol)	0.138	0.004	0.273	**0.044**
Model 3 + adjustment for SHBG	0.139	0.004	0.274	**0.044**
**Estrone**				
Model 1 Crude	0.208	0.081	0.336	**0.002**
Model 2 (adjusted for BMI)	0.167	0.037	0.297	**0.013**
Model 3 (adjusted for BMI, CRP and cholesterol)	0.167	0.034	0.301	**0.014**
Model 3 + adjustment for SHBG	0.181	0.043	0.318	**0.010**
**Estradiol**				
Model 1 Crude	0.170	0.034	0.305	**0.015**
Model 2 (adjusted for BMI)	0.088	-0.067	0.243	0.262
Model 3 (adjusted for BMI, CRP and cholesterol)	0.091	-0.069	0.252	0.263
Model 3 + adjustment for SHBG	0.098	-0.061	0.256	0.224
	**β**	**2.5%CI**	**97.5%CI**	**p**
**Testosterone**				
Model 1 Crude	0.080	-0.052	0.212	0.235
Model 2 (adjusted for WHR)	0.152	0.026	0.278	**0.018**
Model 3 (adjusted for WHR, CRP and cholesterol)	0.157	0.031	0.283	**0.015**
Model 3 + adjustment for SHBG	0.163	0.029	0.296	0.017
**DHEA**				
Model 1 Crude	-0.102	-0.235	0.029	0.127
Model 2 (adjusted for WHR)	-0.084	-0.209	0.042	0.190
Model 3 (adjusted for WHR, CRP and cholesterol)	-0.084	-0.211	0.042	0.187
Model 3 + adjustment for SHBG	-0.071	-0.201	0.059	0.279
**DHT**				
Model 1 Crude	-0.079	-0.212	0.053	0.238
Model 2 (adjusted for WHR)	-0.022	-0.151	0.108	0.742
Model 3 (adjusted for WHR, CRP and cholesterol)	-0.009	-0.141	0.123	0.893
Model 3 + adjustment for SHBG	-0.011	-0.147	0.126	0.878
**Androstenedione**				
Model 1 Crude	0.024	-0.112	0.159	0.730
Model 2 (adjusted for WHR)	0.010	-0.119	0.139	0.879
Model 3 (adjusted for WHR, CRP and cholesterol)	0.010	-0.118	0.140	0.869
Model 3 + adjustment for SHBG	0.013	-0.116	0.143	0.840
**Sex hormone binding globulin**				
Model 1 Crude	-0.064	-0.200	0.073	0.358
Model 2 (adjusted for BMI)	0.017	-0.126	0.161	0.812
Model 3 (adjusted for BMI, CRP and cholesterol)	0.024	-0.121	0.170	0.744

Dependent variable: Log-transformed revised Framingham Stroke Risk Profile. All participants with CRP > 10, participants with previous stroke, and missing information regarding previous stroke were excluded, *n* = 133. The regression coefficient and CI are presented on a standardized SD scale. Specifically, original values have been multiplied by the SD. WHR = waist-to-hip ratio, BMI = body mass index, DHT = dihydrotestosterone, DHEA = dehydroepiandrosterone

High levels of progesterone were significantly associated with high rFSRP in the model adjusting for BMI (β = 0.155 95% CI = 0.025;0.285, *P* = 0.020) and BMI, cholesterol, and CRP (β = 0.150, 95% CI = 0.017;0.283, *P* = 0.027) (Table 2). Similar results were found when analysing the association between 17-α-OH-progesterone and rFSRP (adjusting for BMI: β = 0.146, 95% CI = 0.014; 0.277, *P* = 0.030, and model adjusting for BMI, cholesterol, and CRP β = 0.138, 95% CI = 0.004;0.273, *P* = 0.044). Estrone was significantly positively associated with rFSRP in the crude model (β = 0.208, 95% CI = 0.081; 0.336, *p* = 0.002), when adjusted for BMI, (β = 0.167, 95% CI = 0.037;0.297, *P* = 0.013), and when adjusted for cholesterol and CRP (β = 0.167, 95% CI = 0.034;0.301, *p* = 0.014). Estradiol was significantly associated with the rFRSP in the crude model (β = 0.17, 95% CI = 0.034;0.305, *p* = 0.015), however these associations were no longer significant after adjustments were made in models 2 and 3. Furthermore, testosterone was positively significantly associated with rFRSP in the model adjusting for WHR (β = 0.152, CI = 0.026;0.278, *p* = 0.018) and in the fully adjusted model (β = 0.157, CI = 0.031;0.283, *p* = 0.015). The remaining sex hormones (DHEA, DHT, androstenedione) were not significantly associated with rFSRP in any of the models. After adding adjustments for SHBG in the final model, In the analyses where also SHBG was added to the model, testosterone was still significant (β = 0.160, CI = 0.029;0.292, *p* = 0.017) but non-significant for estradiol, (β = 0.101, CI=-0.063;0.265, *p* = 0.224). No significant associations were found in the linear regression analyses between SHBG and rFSRP.

The bivariate correlation analysis found high correlations between different sex hormones. The hormones with the three highest coefficients were estrone and estradiol (Pearson correlation 0.855, *p* < 0.001), 17-α-OH-progesterone and progesterone (Pearson correlation 0.766, *p* < 0.001) and DHEA and androstenedione (Pearson correlation 0.761, *p* < 0.001).

In the sensitivity analysis, we conducted regression analyses after stratifying for the median age of 64 years old. The effect sizes were similar in the age groups younger or older than 64 years old, except for estradiol that was not significant in the crude model in participants **≥**64 years, and DHEA that was significant only in women **≥**64 of age (supplementary Table 1).

## Discussion

In this study we observed positive significant associations between progesterone, 17-α-OH-progesterone and estrone respectively, and the revised Framingham Stroke Risk Profile in women after menopause. These associations were still significant after adjustments for BMI, CRP, and cholesterol. We also found a significant positive association between testosterone and rFRSP in the adjusted models.

### Progesterone and 17-α-OH-progesterone

Both progesterone and 17-α-OH-progesterone were positively and significantly associated with rFSRP in the models adjusting for BMI, but also when adjusting for BMI, CRP and cholesterol. Large prospective studies investigating the association between endogenous progesterone and stroke in women are limited, especially in postmenopausal women. Our group has previously studied the association between cardiometabolic risk factors and sex hormones in postmenopausal women [18], revealing a positive association between 17-α-OH-progesterone and HOMA-Ir, but a negative association between 17-α-OH-progesterone and BMI. In an observational study investigating both peri- and postmenopausal women, significant positive associations were found in postmenopausal women between both endogenous progesterone (by LC-MS/MS) and 17-α-OH-progesterone respectively, and fasting glucose and also an association between progesterone and HbA_1c_ [20]. Furthermore, in the study by Mezzullo et al. [21] a positive association between 17-α-OH-progesterone and triglycerides was found in postmenopausal women. These results indicate that endogenous progesterone and 17-α-OH-progesterone are associated with cardiometabolic risk factors, in line with the findings in the present study. Besides being fundamental in the female reproductive physiology, progesterone has other effects, among them metabolic effects such as raising insulin levels, and neuroprotective effects after traumatic brain injury [22]. Indeed, previous studies have suggested that progesterone can both promote inflammatory response to cerebrovascular injury but also work as a neuroprotective agent after cerebrovascular injury such as stroke [23]. Experimental studies in rodents have shown that exogenic progesterone can be neuroprotective and decrease the extent of an infarction, possibly by regulating inflammatory actions and mitochondrial functions [5, 23]. However, progesterone levels decrease dramatically during the menopausal transition [21], and derive mainly from precursors from the adrenal gland after menopause [24].

Studies using RIA technique, such as the Swedish longitudinal twin study, revealed that higher endogenous progesterone in women aged 70 years and older was associated with higher serum insulin [25] and increased prevalence of heart failure, even after adjustments were made for CRP, creatinine, and insulin levels [26]. The results are consistent with our study. However, because these studies used the RIA method, the results may be disturbed by inflammatory parameters, besides not being precise enough [27]. Participants in our study had a mean age of 64.5 years old, and our findings suggest that increased levels of endogenous progesterone in this age group are associated with higher estimated stroke risk. The results might have turned out differently in a cohort in which the participants were closer to the menopausal transition.

### Estrone and estradiol

In this study, estrone was positively associated with risk of stroke according to the rFRSP. Estradiol, on the other hand, was only significantly associated with the rFRSP in the crude model and became non-significant after adjustments were made for BMI.

Estrone is both a precursor and a metabolite of estradiol, which is the dominant sex hormone in fertile women. However, after menopause, as the ovarian production of estradiol decreases, the main source for estrone and estradiol are from the adrenal glands, in forms of DHEA, which is converted by aromatase to estrone and estradiol in the peripheral tissues such as adipose tissue [28] making the total adipose tissue an important factor in affecting the levels of hormones. In this study, DHEA was positively associated with rFSRP in the fully adjusted models in the age stratified analyses in women 64 years or older. In the Nurses’ Health Study low concentrations of DHEAS have been associated with greater risk of stroke in postmenopausal women, however, if this association can be explained by an effect on atherosclerosis or is due to a potential damage of the adrenal function is unclear [29]. The association between estrone and cardiovascular diseases has not been as thoroughly investigated as in the case of estradiol. A meta-analysis studying the associations between premature ovarian insufficiency (POI) and ischaemic heart disease, total cardiovascular disease and stroke, found a positive association between POI and ischaemic heart disease and CVD but not between POI and stroke [30]. In another meta-analysis, women undergoing menopause later than 55 years of age had a higher risk of having hemorrhagic stroke compared to women undergoing menopause between 50 and 54 years of age [31].

A recent study using LC-MS/MS and investigating more than 5000 women aged over 70, found a significantly lower risk of major cardiovascular events (both stroke and cardiac events) for participants with estrone concentrations in the second quartile, compared to those in the lowest quartile [32], and furthermore an inverse association between one (DHEA), a precursor of estrone, and major cardiovascular events was found [32]. In a case-control study of postmenopausal women there was no association between the odds of atherosclerosis and increasing quartiles of estrone (measured by RIA) [33]. However, as stated above, measurements with RIA are not precise enough when measuring low-range sex hormones, and the results may also be interfered with by inflammatory parameters [27]. The potential biological mechanisms behind these associations are not entirely understood. Estradiol is indeed a known prothrombotic hormone both when administered as an oral contraceptive, and when naturally elevations of endogenous estradiol occur such as in pregnancy [34]. There is also evidence that estradiol associates with worsening of glucose homeostasis and development of diabetes mellitus, however, even though many of these studies are not using LC-MS/MS, they still indicate that higher estradiol levels in postmenopausal women may have a negative effect on their cardiometabolic health [35].

Given the close relationship between estradiol and estrone in steroid biosynthesis, there was, as expected, a high correlation between estrone and estradiol (Pearson’s correlation 0.86). Even so, we were not able to find a significant association between estradiol and rFSRP after adjustments were made for BMI, indicating that adipose tissue is a strong confounder in this association. The additional analysis including the SHGB did not change the association in the fully adjusted model. The association between estradiol and stroke has further been investigated in a meta-analysis, in which no associations were found between estradiol and stroke in postmenopausal women [36], even though estradiol has shown positive associations with coronary heart disease [37] and stroke risk [38] in women after menopause.

### Testosterone

Our results show that among the androgens, testosterone was positively associated with the rFRSP in the fully adjusted model. In a previous study of this cohort [18], testosterone was significantly positively associated with age, which is incorporated in the algorithm of the rFSRP, and age may thus be an important driver of the association found between testosterone and rFSRP. Conflicting results have been published with regard to the association between testosterone and cardiovascular morbidity and mortality in women [39, 40]. The MESA study reported similar results to our study, with a positive association between testosterone and incident cardiovascular disease [41], as did Benn et al., showing that extreme values (above the 90th percentile) in testosterone were associated with higher incidence of CVD [42]. Another study found no associations between testosterone and stroke risk [38]. However, both these studies defined levels of testosterone based on RIA technique, and are therefore less reliable than studies using LC-MS/MS. Yet another study using LC-MS/MS found no association between testosterone levels and ischaemic stroke in a case-control study in post-menopausal women within the Nurse’s health study [43]. In a more recent study, Islam et al. investigated the association between sex hormones, measured by LC-MS/MS, and major adverse cardiovascular events (MACE) in women above 70 years old, and found inverse associations between both testosterone and MACE after approximately 4.4 years of follow-up [32]. On the other hand, Meun et al. found no associations between DHEA or testosterone and incident stroke or coronary heart disease after 11 years of follow-up in the Rotterdam study [44]. Even though these studies are not completely comparable– partly due to different outcomes and age groups included– the results may be described as conflicting. The biological mechanisms behind these associations are still to be elucidated. After menopause, the main source of testosterone is from conversion in peripheral tissues from adrenal or ovarian androstenedione, but there is also a smaller production of testosterone by the ovaries and the adrenal gland [45, 46]It is known that women with hyperandrogenic states such as polycystic ovary syndrome, have a higher risk of developing cardiovascular disease including stroke, but for postmenopausal women this correlation seems to be driven mainly by obesity [47]. In our study, however, the adjustment for WHR, as a measure of central obesity, did reveal a significant association between testosterone and rFSRP, emphasizing the influence of body shape. The effect of testosterone is also affected by its binding to SHBG, however in the analyses using adding SHBG to the fully adjusted model, the significance of the association between testosterone and rFSRP remained. It has been suggested that androgens have immune-modulating effects and ability to the suppress activation of pro-inflammatory cytokines, possibly having an impact on atherosclerosis process [48], but further studies are needed to fully understand these mechanisms.

### Strengths and limitations

One strength of this study is its robust study protocol, with reliable assessment of variables and covariates included in the rFSRP. Another strength is the measurement of sex hormones by LC-MS/MS, which is the gold standard when measuring steroid hormones at low concentrations, as in our group of postmenopausal women.

Using frozen sera may have altered the levels of steroids, but recent research shows that there is excellent reproducibility in steroid measurements by LC-MS/MS even when serum has been frozen at -80° C for 10 years [49].

Our study is an exploratory study with the aim to find associations between sex hormones and the estimated risk of developing stroke by utilizing available data material. Our way of performing the analyses confers a risk of type 1 errors due to multiple testing. The findings need to be interpreted with that in mind.

The use of a risk score algorithm is an estimation, and it would have been preferable to use stroke events as outcome. However, in this small group consisting of mainly healthy women, the event rate is low, and the cohort size would have needed to be considerably larger. Even though we have adjusted for known confounders, there is a possibility that there may be residual confounding affecting the results. We did not have access to information on certain diagnoses and lifestyle factors, such as polycystic ovary syndrome, or thyroid function, dietary habits or environmental factors, which could have resulted in residual confounding. We did not have information on estrogen receptor status, which may have led to residual confounding, given that different receptor dominance are associated with different cardiovascular outcomes [50] Also, the small sample size is a limitation to the study and calls for cautiousness in the number of included covariates, and the results need to be interpreted with the sample size in mind. In a previous study of this cohort we have shown which cardiometabolic variables associate with the eight included hormones [18]. Among the hormones associated with rFSRP, testosterone was also positively associated with age, which is incorporated in the algorithm of the rFSRP. For the other hormones (progesterone, 17-α-OH-progesterone, and estrone) there were no individual significant associations with the other components of the rFSRP. In this study we did not adjust for age. To our knowledge, few studies have investigated the association between sex hormones and Framingham risk score, however, the study by Chock et al. is similar to ours, and did not adjust for age [51]. Moreover, in the present study, sensitivity analyses were performed using the median age 64 years, showing similar results of the estimates in both age groups. Even so, since testosterone was significantly associated with age, and age is incorporated in the algorithm, it is likely that age is one of the most important variables driving the association between testosterone and rFSRP in this cohort. In the previous study of this cohort [18], we found no significant associations between physical activity or alcohol consumption and estrone or estradiol, therefore we chose not to adjust for these covariates. Since we did not have information on menopausal status, we chose to include only women with age > 54 and with estradiol concentrations that are low (< 20 pg/ml), but we were not able to adjust for time since menopause.

There may also be non-linear associations that are complex to explore in smaller cohorts, therefore, larger prospective studies with stroke events as a outcome, are warranted to explore the associations and to understand the biological mechanisms behind them, and to better understand the role of sex hormones in the cardiometabolic health of postmenopausal women.

## Conclusions

Positive associations were found between levels of testosterone, estrone and progesterone and estimated 10-year risk of stroke, according to the revised Framingham Stroke Risk Profile in postmenopausal women. These findings are important in the research of sex hormones as potential biomarkers for cardiovascular disease and adds knowledge to previous studies on the area. Prospective larger studies including information on cardiovascular events are warranted to confirm these results and for better understanding on the mechanisms and pathways behind the role of sex hormone levels in the cardiovascular health of postmenopausal women.

## Electronic supplementary material

Below is the link to the electronic supplementary material.

Supplementary Material 1

## Data Availability

The datasets generated and/or analysed during the current study are not publicly available due privacy regulations but are available from the corresponding author on reasonable request.
