# Supplementary material for: Endogenous sex hormone levels are associated with the revised Framingham Stroke Risk Profile in postmenopausal women: a longitudinal study in a Swedish cohort
Source: BMC Endocr Disord. 2025 Jan 26;25:24. doi: 10.1186/s12902-025-01841-3 (PMC11765893; doi:10.1186/s12902-025-01841-3)
Supplement: Supplementary file 1 — Supplementary Material 1 [file 12902_2025_1841_MOESM1_ESM.docx]

| Supplementary table 1. Showing the results from the sensitivity analysis stratifying for median age 64. | | | | | | | | | | |
| --- | --- | --- | --- | --- | --- | --- | --- | --- | --- | --- |
|  | **Age <64 N=70** | | | | | | **Age ≥64 N=63** | | | |
|  | | **β** | **2.5%CI** | **97.5%CI** | p |  | **β** | **2.5%CI** | **97.5%CI** | p |
| **Progesterone** | |  |  |  |  |  |  |  |  |  |
| Model 1 Crude | | -0.031 | -0.184 | 0.121 | 0.683 |  | 0.010 | -0.078 | 0.097 | 0.825 |
| Model 2 (adjusted for BMI) | | 0.016 | -0.136 | 0.169 | 0.830 |  | 0.029 | -0.063 | 0.121 | 0.532 |
| Model 3 (adjusted for BMI, CRP and cholesterol) | | 0.016 | -0.137 | 0.170 | 0.833 |  | 0.032 | -0.064 | 0.127 | 0.512 |
| **17-α-OH-progesterone** | |  |  |  |  |  |  |  |  |  |
| Model 1 Crude | | 0.043 | -0.078 | 0.163 | 0.482 |  | 0.027 | -0.074 | 0.129 | 0.592 |
| Model 2 (adjusted for BMI) | | 0.065 | -0.051 | 0.181 | 0.269 |  | 0.040 | -0.063 | 0.143 | 0.436 |
| Model 3 (adjusted for BMI, CRP and cholesterol) | | 0.059 | -0.059 | 0.177 | 0.324 |  | 0.044 | -0.064 | 0.153 | 0.417 |
| **Estrone** | |  |  |  |  |  |  |  |  |  |
| Model 1 Crude | | 0.107 | -0.013 | 0.228 | 0.079 |  | 0.081 | -0.016 | 0.179 | 0.100 |
| Model 2 (adjusted for BMI) | | 0.076 | -0.045 | 0.197 | 0.214 |  | 0.072 | -0.030 | 0.173 | 0.162 |
| Model 3 (adjusted for BMI, CRP and cholesterol) | | 0.061 | -0.065 | 0.187 | 0.337 |  | 0.077 | -0.026 | 0.181 | 0.140 |
| **Estradiol** | |  |  |  |  |  |  |  |  |  |
| Model 1 Crude | | 0.123 | 0.007 | 0.239 | 0.037 |  | 0.050 | -0.049 | 0.149 | 0.319 |
| Model 2 (adjusted for BMI) | | 0.068 | -0.064 | 0.200 | 0.306 |  | 0.029 | -0.084 | 0.143 | 0.608 |
| Model 3 (adjusted for BMI, CRP and cholesterol) | | 0.051 | -0.087 | 0.189 | 0.465 |  | 0.036 | -0.083 | 0.155 | 0.549 |
| **Testosteron** | |  |  |  |  |  |  |  |  |  |
| Model 1 Crude | | 0.052 | -0.065 | 0.170 | 0.377 |  | 0.033 | -0.069 | 0.136 | 0.520 |
| Model 2 (adjusted for WHR) | | 0.072 | -0.043 | 0.187 | 0.215 |  | 0.067 | -0.043 | 0.176 | 0.229 |
| Model 3 (adjusted for WHR, CRP and cholesterol) | | 0.078 | -0.037 | 0.192 | 0.181 |  | 0.065 | -0.047 | 0.178 | 0.250 |
| **DHEA** | |  |  |  |  |  |  |  |  |  |
| Model 1 Crude | | 0.031 | -0.067 | 0.130 | 0.528 |  | 0.126 | -0.012 | 0.264 | 0.072 |
| Model 2 (adjusted for WHR) | | 0.017 | -0.080 | 0.114 | 0.735 |  | 0.154 | 0.014 | 0.293 | 0.032 |
| Model 3 (adjusted for WHR, CRP and cholesterol) | | 0.026 | -0.071 | 0.124 | 0.593 |  | 0.167 | 0.023 | 0.311 | 0.023 |
| **DHT** | |  |  |  |  |  |  |  |  |  |
| Model 1 Crude | | 0.017 | -0.083 | 0.118 | 0.732 |  | 0.042 | -0.084 | 0.167 | 0.509 |
| Model 2 (adjusted for WHR) | | 0.030 | -0.069 | 0.129 | 0.550 |  | 0.072 | -0.059 | 0.202 | 0.277 |
| Model 3 (adjusted for WHR, CRP and cholesterol) | | 0.039 | -0.061 | 0.139 | 0.437 |  | 0.068 | -0.065 | 0.202 | 0.309 |
| **Androstenedione** | |  |  |  |  |  |  |  |  |  |
| Model 1 Crude | | 0.036 | -0.068 | 0.140 | 0.493 |  | 0.068 | -0.045 | 0.181 | 0.233 |
| Model 2 (adjusted for WHR) | | 0.019 | -0.084 | 0.123 | 0.710 |  | 0.072 | -0.041 | 0.184 | 0.208 |
| Model 3 (adjusted for WHR, CRP and cholesterol) | | 0.025 | -0.079 | 0.128 | 0.632 |  | 0.071 | -0.044 | 0.185 | 0.222 |
| Dependent variable: Log-transformed revised Framingham Stroke Risk Profile. All participants with CRP>10, participants with previous stroke, and missing information regarding previous stroke were excluded.  The regression coefficient and CI are presented on a standardized SD scale. Specifically, original values have been multiplied by the SD. WHR= waist-to-hip ratio, BMI=body mass index, DHT=dihydrotestosterone, DHEA=dehydroepiandrosterone | | | | | | | | | | |
